# Refinement of the critical genomic region for congenital hyperinsulinism in the Chromosome 9p deletion syndrome

**DOI:** 10.12688/wellcomeopenres.15465.2

**Published:** 2020-08-04

**Authors:** Indraneel Banerjee, Senthil Senniappan, Thomas W. Laver, Richard Caswell, Martin Zenker, Klaus Mohnike, Tim Cheetham, Matthew N. Wakeling, Dunia Ismail, Belinda Lennerz, Miranda Splitt, Merih Berberoğlu, Susann Empting, Martin Wabitsch, Simone Pötzsch, Pratik Shah, Zeynep Siklar, Charles F. Verge, Michael N. Weedon, Sian Ellard, Khalid Hussain, Sarah E. Flanagan

**Affiliations:** 1 Department of Paediatric Endocrinology, Royal Manchester Children's Hospital, Manchester, UK; 2 Department of Paediatric Endocrinology, Alder Hey Children's NHS Foundation Trust, Liverpool, UK; 3 Institute of Biomedical and Clinical Science, University of Exeter Medical School, Exeter, UK; 4 Institute of Human Genetics, University Hospital, Otto-von-Guericke University, Magdeburg, Germany; 5 Department of Paediatrics, University Hospital, Otto-von-Guericke University, Magdeburg, Germany; 6 Department of Paediatric Endocrinology, Royal Victoria Infirmary, Newcastle, UK; 7 Department of Paediatric Endocrinology & Diabetes, Royal Alexandra Children’s Hospital, Brighton, UK; 8 Department of Paediatrics and Adolescent Medicine, Ulm University Hospital, Ulm, Germany; 9 Northern Genetics Service, Newcastle upon Tyne Hospitals NHS Foundation Trust, Newcastle upon Tyne, UK; 10 Department of Pediatric Endocrinology, Ankara University School of Medicine, Ankara, Turkey; 11 Department for Children and Adolescent Medicine, HELIOS Vogtland-Klinikum Plauen, Plauen, Germany; 12 Endocrinology Department, Great Ormond Street Hospital for Children NHS Foundation Trust, London, UK; 13 Department of Endocrinology, Sydney Children's Hospital, Randwick and School of Women's and Children's Health,, Sydney, New South Wales, Australia; 14 Department of Pediatric Medicine, Sidra Medicine, Doha, Qatar

**Keywords:** Chromosome 9p, Deletions, Hyperinsulinism, Hypoglycaemia

## Abstract

**Background: **Large contiguous gene deletions at the distal end of the short arm of chromosome 9 result in the complex multi-organ condition chromosome 9p deletion syndrome.  A range of clinical features can result from these deletions with the most common being facial dysmorphisms and neurological impairment. Congenital hyperinsulinism is a rarely reported feature of the syndrome with the genetic mechanism for the dysregulated insulin secretion being unknown.

**Methods: **We studied the clinical and genetic characteristics of 12 individuals with chromosome 9p deletions who had a history of neonatal hypoglycaemia. Using off-target reads generated from targeted next-generation sequencing of the genes known to cause hyperinsulinaemic hypoglycaemia (n=9), or microarray analysis (n=3), we mapped the minimal shared deleted region on chromosome 9 in this cohort. Targeted sequencing was performed in three patients to search for a recessive mutation unmasked by the deletion.

**Results: **In 10/12 patients with hypoglycaemia, hyperinsulinism was confirmed biochemically. A range of extra-pancreatic features were also reported in these patients consistent with the diagnosis of the Chromosome 9p deletion syndrome. The minimal deleted region was mapped to 7.2 Mb, encompassing 38 protein-coding genes. *In silico* analysis of these genes highlighted *SMARCA2* and *RFX3* as potential candidates for the hypoglycaemia. Targeted sequencing performed on three of the patients did not identify a second disease-causing variant within the minimal deleted region.

**Conclusions: **This study identifies 9p deletions as an important cause of hyperinsulinaemic hypoglycaemia and increases the number of cases reported with 9p deletions and hypoglycaemia to 15 making this a more common feature of the syndrome than previously appreciated.  Whilst the precise genetic mechanism of the dysregulated insulin secretion could not be determined in these patients, mapping the deletion breakpoints highlighted potential candidate genes for hypoglycaemia within the deleted region.

## Introduction

Monosomy of part of the short arm of chromosome 9 causes the complex congenital condition chromosome 9p deletion syndrome (MIM: 158170) ^1^. These large contiguous gene deletions can occur in isolation or form part of an unbalanced translocation ^2^. The cardinal clinical features of the 9p deletion syndrome are craniofacial dysmorphisms, including trigonocephaly, midface hypoplasia, flat nasal ridge, long philtrum, short neck and developmental delay. Other common features include musculo-skeletal abnormalities, congenital heart defects, abdominal wall defects and disorders of sexual differentiation ^3^. A further rare feature is hypoglycaemia, which has been described in 3 of the >100 genetically confirmed cases ^4– 6^.

The phenotypic heterogeneity observed between individuals with the chromosome 9p deletion syndrome is likely to reflect differences in the extent of the deletion, with individual features resulting from haploinsufficiency of a specific gene(s). An example is seen in males with 46,XY gonadal dysgenesis (MIM: 154230) which has been linked to disruption of the putative sex-determining genes *DMRT1* and *DMRT2* on 9p ^7^.

Recent efforts have focussed on defining the critical region for the 9p deletion syndrome but there have been some differences in results. Swinkels *et al.* refined the critical region to a 300 kb stretch of DNA on 9p22.3; however, this region did not overlap with the critical region mapped by Faas *et al.* ^3, 8^. Given the differences in the craniofacial features between the cohorts reported it seems likely that there is not a single ‘critical region’ for the 9p deletion syndrome but rather that the syndrome represents a phenotypically and genetically heterogeneous group of disorders with the extent of the deletion, and in some cases the reciprocal trisomy, determining the phenotype.

Congenital hyperinsulinism is a rare condition of hypoglycaemia due to dysregulated insulin production from pancreatic beta cells ^9^. Despite major advances in genetics the underlying cause of congenital hyperinsulinism is not identified in approximately 55% of patients ^10^. Studying patients with congenital hyperinsulinism and the 9p deletion syndrome provides an opportunity to further unravel the genetic underpinnings of dysregulated insulin secretion in congenital hyperinsulinism.

In this study we investigated the clinical and genetic characteristics of 12 patients with congenital hypoglycaemia and a large deletion on chromosome 9p. We mapped the genomic breakpoints in all 12 patients which allowed for refinement of the critical region for hypoglycaemia to 7.2 Mb encompassing 38 genes. We sought to identify candidate genes for congenital hyperinsulinism in this region, an approach which has been successfully employed for gene discovery in other conditions ^11^.

## Methods

### Cohort

A total of 12 patients with large deletions of the short arm of chromosome 9 were identified (as described below) following referral for genetic testing for congenital hyperinsulinism or a history of neonatal hypoglycaemia. Informed consent for publication of the patients’ details was obtained. This study was approved by the North Wales Research Ethics Committee (517/WA/0327).

The DECIPHER database was searched for individuals who had hypoglycaemia and deletions of chromosome 9p which overlapped with the deletions identified in our cohort ^6^.

### Calling deletions

In nine patients multiple syndromic features had prompted microarray analysis leading to the identification of a 9p deletion. In the remaining three patients a deletion on 9p was detected using SavvyCNV (release 1) using off-target reads from the next-generation sequencing analysis of the known congenital hyperinsulinism genes. This technique calls 97.5% of true CNVs >1Mb ^12^.

Break points were mapped in patients 1–9 using off-target reads from the targeted next generation sequencing data. In these patients analysis of the known hyperinsulinism genes did not identify a mutation. In patients 10–12 the breakpoints were mapped by microarray analysis, DNA was not available for targeted sequencing in these individuals. In 5/11 patients the 9p deletion formed part of an unbalanced translocation ( Table 1).

**Table 1.** Clinical and genetic characteristics of patients with Chromosome 9p deletion syndrome. PDA =Patent Ductus Arteriosus, VSD = Ventricular Septal Defect, ASD = Atrial Septal Defect, PFO = Patent Foramen Ovale. *Patient 10 and Patient 11 are siblings. ^#^ Genomic coordinates (GRCh37/hg19) of copy number variant detected by analysis of tNGS off-target reads (patients 1–9) or microarray analysis (patients 10–12).

	Patient 1	Patient 2	Patient 3	Patient 4	Patient 5	Patient 6	Patient 7	Patient 8	Patient 9	Patient 10	Patient 11 *	Patient 12
**Karyotype**	46,XY, del(9)(p22.1)	Not performed	46,XX,del(9) (p22.2)	46,XY,der(9)t(9;1 3)(p24;q22.3)	46,XX,del(9) (p23).ish del(9)(pter-)	46,XX,der(9)t(8;9) (q24.1;p24)	Not performed	Not performed	46,XX,del(9) (p24.1)	46,XY,der(9)t(9;13)(p 23;q33.3)	46,XY,der(9)t(9;13) (p23;q33.3)	46,XY,del(9) (p23)
**Chromosome** **9p deletion ^#^**	0-19,200,000	0-14,000,000	0-17,600,000	0-9,806,011	0-9,330,617	0-7,800,000	0-7,200,000	0-7,600,000	0-7,600,000	0-12,450,000	0-12,450,000	0-10,955,813
**Reciprocal** **Duplication ^#^**	None detected	None detected	None detected	Chr13:75000000- 115200000	None detected	Chr8:117800000- 146400000	Chr7:0- 10,000,000	None detected	None detected	Chr13:107,452,410- 115,105,270	Chr13:107,452,410- 115,105,270	None detected
**Gender**	Male	Male	Female	Assigned Female	Female	Female	Female	Male	Female	Male	Male	Male
**Birthweight (g)**	4478	3440	3200	2960	3520	3670	3510	3000	1700	3650	4160	5050
**Gestation** **(wks)**	38	38	40	37	38	40	39	37	34	38+1	41	42
**Birth weight** **SDS**	2.87	0.86	-0.10	0.16	1.32	0.99	0.93	0.26	-1.22	1.27	1.49	2.93
**Current Age** **(yrs)**	3	2	2	10	8	9	7	8	2	11	20	8
**Hyperinsulinaemic hypoglycaemia**												
**Age at onset**	3 days	2 days	20 weeks	Birth	Birth	Birth	Birth	8 weeks	Birth	Birth	Birth	Birth
**Age at** **remission**	26 days	43 weeks	7 months	4 years	1.4 years	1.3 yrs	6 weeks	Ongoing at 8 yrs	Ongoing at 2.8yrs	Ongoing	Ongoing	1 week (no follow-up)
**Blood glucose** **at onset of** **hypoglycaemia** **(mmol/L)**	2.9	1.8	2.5	1.4	1.8	1.8	1.4	2.7	2.0	0.7	1.2	1.9
**Insulin** **(pmol/L)**	97	28	<6.0	111	96	42	51	47	53	100	190	Not tested
**C-Peptide** **(pmol/L)**	228	Not tested	Not tested	980	540	Not tested	Not tested	Not tested	364	629	Not tested	Not tested
**Treatment** **details**	Diazoxide until remission (dose not available)	Diazoxide 10.5 mg/ kg/day	No treatment	Diazoxide 5 mg/kg/day until remission	Diazoxide 7mg/kg/day until remission	Diazoxide until remission (dose not available)	Diazoxide 10mg/kg/day until remission	Diazoxide 2mg/kg/day ongoing	Diazoxide 6mg/kg/day	Diazoxide 10mg/kg/day ongoing	Diazoxide 10mg/kg/day ongoing	No treatment
**Extra-pancreatic features**												
**Facial** **dysmorphism**	Metopic suture defect Hypotelorism High arched palate Small low set ears	Not noted in infancy	Metopic suture synostosis Low set ears High narrow palate Hypotelorism	Metopic prominence Bi-temporal narrowing Prominent eyes Low set ears Long philtrum Micrognathia	Broad forehead Brachycephaly Low set ears	Microcephaly Up-slanting palpebral fissures High nasal bridge Large mouth Thin upper lip	Sub mucosal cleft palate Uvula bifida Deep-seated ears Hypertelorism Small chin	None noted	Prominent forehead Flat mid face Wide base gait Low set ears	Macroglossia Small deep-seated ears	Macroglossia Small deep-seated ears	Macroglossia
**Digits**	Wide sandal gap	Normal	Long fingers and toes	4 limb postaxial polydactyly Syndactyly 2 ^nd^ and 3 ^rd^ toes	Unilateral clinodactyly Bilateral sandal gap	Broad and long fingers and toes	Normal	Normal	Broad fingers	Normal	Normal	Normal
**Cardiac**	VSD	PDA	ASD	ASD, PDA	ASD, PDA	VSD	PDA, PFO/ASD II	-	-	-	-	-
**Other features**	Abdominal wall hernia Hypospadia, Micropenis Large rockerbottom feet Telangiectasia	Delayed expressive speech Mild gross motor delay (walked at 18 months) Autistic features	Moderate global developmental delay Gastrooesophageal reflux Gastrostomy fed Obstructive sleep apnoea Moderate bilateral conductive hearing loss Nystagmus with normal vision acuity	Severe under virilisation with dysgenetic testes Perineal hypospadias Widely spaced nipples Congenital bilateral glaucoma Abnormal corpus callosum Small cerebellum Severe developmental delay Large CSF spaces Slow weight gain	Tracheo- oesophageal fistula	Widely spaced nipples Skeletal dysplasia Seizures	Haemorrhages: cortical, subcortical cerebellar and brain marrow Significant developmental delay	Glandular hypospadia Developmental delay. Cranial MRI: Occipital encephalomalasia and perifocal gliosis	Developmental delay (motor and speech)	Developmental delay (motor and speech) Lymphedema in both legs Hepatomegaly Small umbilical hernia	Developmental delay (motor and speech) Muscular hypotension Hepatomegaly Umbilical hernia	Global developmental delay (motor and speech). MRI showed delayed myelination Cryptorchidism

### Sequencing of the deleted region

To search for recessive mutations unmasked by the deletion, next generation sequencing was performed in three patients following targeted capture of the Chr9p24 region (patients 4, 5 and 6, Table 1). Illumina-compatible libraries were prepared after fragmentation of genomic DNA to ^~^200bp average size, then enriched for target regions using a custom RNA bait library designed against chr9:1-7,834,443 (GRCh37/hg19) with medium stringency against repetitive sequences (Prognosys Biosciences Inc., formerly of La Jolla, CA). Hybridization, capture, washing and amplification (15 cycles) were performed using a Rivia Targeted Enrichment Kit according to the manufacturer’s instructions (Rivia, formerly of La Jolla, CA). Libraries were sequenced on an Illumina HiSeq 2000 using 100 base paired-end reads.

Sequence data was analysed using an approach based on the GATK best practice guidelines ^13^. Reads were aligned to the GRCh37/hg19 human reference genome with BWA mem (version 0.7.15) ^14^ followed by local re-alignment using GATK IndelRealigner (version 3.7.0) ^15^. Large sections of the region are low complexity and while mean target coverage was 36X, 34X and 41X, only 56%, 56% and 58% of the minimal deleted region was covered at 10X or above in the three samples, respectively. Variants were called using GATK haplotype caller and annotated using Alamut Batch (Interactive Biosoftware version 1.11, Rouen, France) (an open-access equivalent is ANNOVAR). ^16^. We excluded variants present in gnomAD ^17^ at a frequency greater than 1 in 27,000 - the highest published prevalence of hyperinsulinism in an outbred population ^18^. Variants that were homozygous in internal controls (n = 65) and intronic variants that were not predicted to affect splicing by the *in silico* tools MaxEntScan ^19^, SpliceSiteFinder-like ^20^, and NNSPLICE ^21^ were excluded.

### Evaluation of protein expression of candidate genes in deleted region

The expression of genes within the deleted region was assessed by the median transcripts per million value from the Genotype-Tissue Expression (GTEx) portal.

## Results

### Clinical characteristics

Clinical characteristics of the cohort are provided in Table 1. Hypoglycaemia (blood glucose <3.0 mmol/l) was diagnosed in 9/12 patients at birth and in three patients at the age of 3 days, 8 weeks and 20 weeks respectively. In all patients cortisol deficiency was excluded clinically and biochemically at diagnosis and no patients had evidence of growth failure. In 10/12 patient’s in our cohort and one patient reported in the literature ^4^ detectable insulin at the time of hypoglycaemia confirmed a diagnosis of congenital hyperinsulinism which was treated with diazoxide. In the four remaining patients, two from our cohort (patients 3 and 12) and two from the literature, a diagnosis of congenital hyperinsulinism was not confirmed. In three of these patients insulin was either not measured or the results were not reported (patient 12, reference 5 and https://decipher.sanger.ac.uk/patient/249708). In the final patient (patient 3) insulin was measured at the time of hypoglycaemia but was suppressed (less than 6.0 pmol/L). The duration of hypoglycaemia varied considerably within our cohort with one child having transitory hypoglycaemia not requiring treatment yet another patient requiring ongoing diazoxide treatment at 8 years.

Two patients within the cohort (patients 10 and 11) were affected siblings; the remaining 10 patients were unrelated and had no family history of hypoglycaemia. Extra-pancreatic features previously reported in patients with Chromosome 9p deletions were observed in all individuals although there was no uniform phenotype. Common features reported in our cohort include cardiac anatomical defects in seven patients, facial dysmorphism in ten patients, digit/limb abnormalities in six patients and undervirilisation in four patients ( Table 1).

### The minimal deleted region for hypoglycaemia is 7.2 Mb

**Figure 1.**
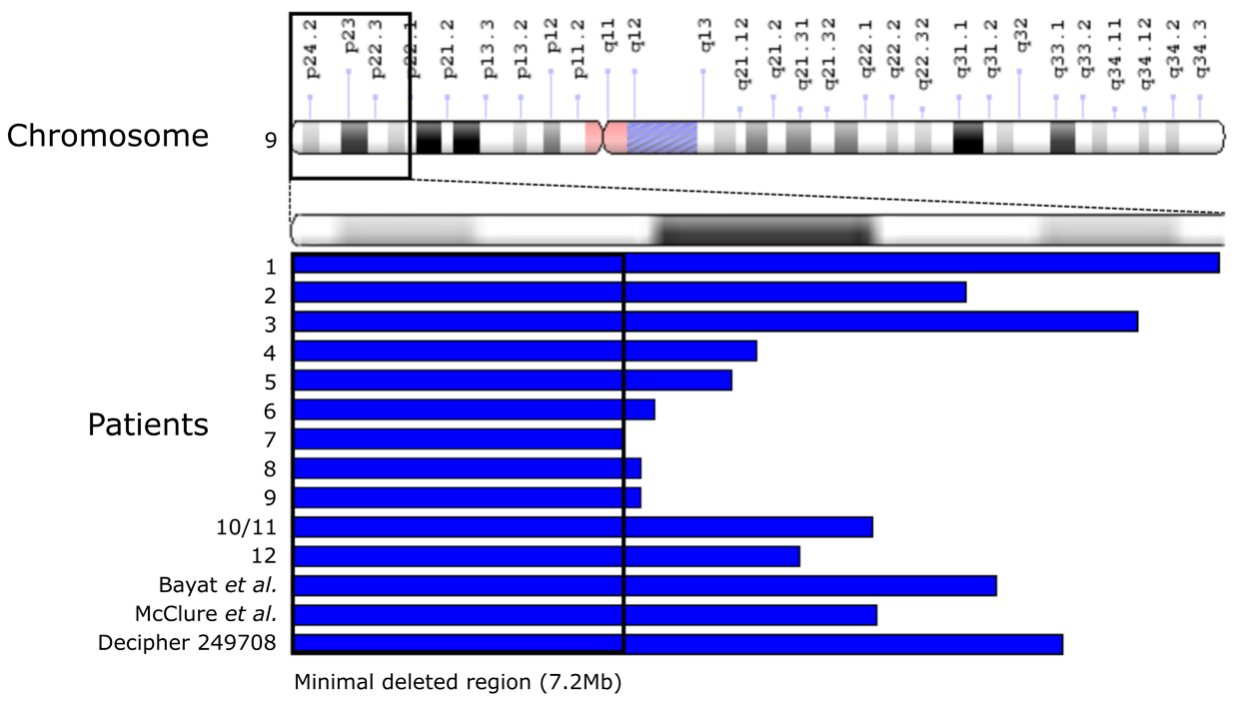
Diagram showing the deletions in our patients (patients 10 and 11 are siblings) and three other reported patients with 9p deletions and hypoglycaemia. Details of the size of the individual deletions are provided in Table 1. A list of the NCBI RefSeq genes within the 7.2Mb minimal deleted region is provided in Table 2.

Analysis of sequence data confirmed deletions on chromosome 9p which ranged in size from 7.2 Mb to 19.2 Mb. These were aligned and compared to the deletions identified in the two patients reported in the literature and an individual listed on the DECIPHER database with a 9p deletion and hypoglycaemia ^4, 5^ ( https://decipher.sanger.ac.uk/patient/249708). The minimal deleted region shared between the 15 patients spanned 7.2Mb (Chr9:0-7200000[hg19], 9p24.3-9p24.1) ( Figure 1).

### The minimal deleted region for hypoglycaemia includes 38 genes

The 7.2Mb minimal deleted region on Chromosome 9 contains 38 protein-coding NCBI RefSeq genes ( Table 2). Of these, *SMARCA2*, *RFX3*, *CDC37L1* and *UHRF2* have a gnomAD pLI score of >0.9 indicating that they are intolerant to loss-of-function variants ^17^. The three genes with the highest levels of expression in the pancreas are *AK3*, *SMARCA2* and *VLDLR* all with a median transcripts per million value of >8 on the Genotype-Tissue Expression (GTEx) portal. Three further genes ( *KANK1*, *RFX3* and *JAK2*) are involved in pathways associated with insulin regulation according to the UniProt gene ontology database ^22^.

**Table 2.** Data on the genes within the minimal deleted region (Chr9:0-7200000[hg19], 9p24.3-9p24.1). pLI scores were obtained from gnomAD. Pancreatic expression was obtained from the Genotype-Tissue Expression (GTEx) portal (gtexportal.org). NA indicates the gene was not found in this database. When disease-causing mutations have been reported details of the associated syndrome and the inheritance of mutations are provided.

NCBI RefSeq Gene	gnomAD pLI	Pancreatic expression GTEx (median transcripts per million)	Disease-causing gene OMIM ID (Phenotype, Inheritance)
*WASHC1*	NA	NA	-
*FOXD4*	0	0.27	-
*CBWD1*	0	1.705	-
*DOCK8*	0	1.79	# 243700 (Hyper-IgE recurrent infection syndrome. Recessive)
*KANK1*	0	8.72	# 612900 (Cerebral palsy, spastic quadriplegic, 2)
*DMRT1*	0.74	NA	-
*DMRT3*	0	0	-
*DMRT2*	0.01	0.02	-
*SMARCA2*	1	13.4	# 601358 (Nicolaides- Baraitser syndrome. Dominant)
*VLDLR*	0	8.84	* 192977 (Cerebellar hypoplasia and mental retardation with or without quadrupedal locomotion 1. Dominant)
*KCNV2*	0	0.07	* 607604 (Retinal cone dystrophy 3B. Dominant)
*PUM3*	NA	NA	
*RFX3*	1	1.18	
*GLIS3*	0	3.52	# 610199 (Diabetes mellitus, neonatal, with congenital hypothyroidism, Recessive)
*SLC1A1*	0	1.47	# 222730 (Dicarboxylic aminoaciduria, Recessive)
*SPATA6L*	0	2.04	-
*CDC37L1*	0.97	6.83	-
*PLPP6*	NA	NA	-
*AK3*	0	24.6	-
*RCL1*	0.06	6.52	-
*JAK2*	0.65	2.43	# 600880 (Budd-Chiari syndrome, Somatic)
*INSL6*	0	0	-
*INSL4*	0	NA	-
*RLN2*	0	0.151	-
*RLN1*	0.01	0.0404	-
*PLGRKT*	0	5.03	-
*CD274*	0.2	1.2	-
*PDCD1LG2*	0	0.167	-
*RIC1*	NA	NA	# 618761 (Catifa Syndrome, Recessive)
*ERMP1*	0	4.7	-
*MLANA*	0	0.0957	-
*KIAA2026*	0.66	4.04	-
*RANBP6*	0	5.44	-
*IL33*	0	1.57	-
*TPD52L3*	0.2	0	-
*UHRF2*	1	6.91	-
*GLDC*	0	0.0397	# 605899 (Glycine encephalopathy, Recessive)
*KDM4C*	0	5.15	-

To test whether the deletion was unmasking a second recessively inherited mutation on the opposite allele we performed targeted capture followed by next generation sequencing of the minimal deleted region in three unrelated individuals. No rare variants shared by all three samples were identified. We also searched for genes harbouring different rare variants in each of the three samples but did not identify any genes which met this criterion.

## Discussion

Our cohort of 12 patients with 9p deletions and hypoglycaemia is the largest reported series and significantly widens the phenotypic spectrum over and above the three reported cases. In 10 of the 12 patients congenital hyperinsulinism was confirmed, whilst in two patients insulin was either not measured at the time of hypoglycaemia or was shown to be appropriately suppressed. Variability in extra-pancreatic phenotypes was observed in our cohort with specific features likely to be determined by the extent of the deletion in each patient and in five cases the reciprocal trisomy. This study allowed the refinement of the critical region for the Chromosome 9p deletion syndrome which features hypoglycaemia to 7.2 Mb. The critical gene(s)/regulatory region(s) within this locus are not known.

As insulin was appropriately suppressed in one patient in our cohort and was not measured in three further individuals we cannot be certain that hypoglycaemia results from dysregulated insulin secretion in all cases with a 9p deletion. If these four patients do have a different mechanism for hypoglycaemia compared to the congenital hyperinsulinism group, we would, however, not expect the size of the minimal deleted region for congenital hyperinsulinism calculated in this report to change given that the deletions in these patients were not critical for determining the boundaries on the 7.2 Mb region ( Table 1 and Figure 1).

There are four possible mechanisms by which large deletions can cause disease: 1) disruption of a gene at the breakpoint 2) haploinsufficiency of a gene within the deletion 3) unmasking a recessive mutation in a gene within the deleted region and 4) disruption of an imprinted gene. None of the genes within the deleted region are known to be imprinted and the breakpoints for the deletions varied between patients in our cohort, making it unlikely that the disruption of a gene at a breakpoint is the cause of the hypoglycaemia in these patients. We performed targeted sequencing of the minimal deleted region to search for recessive mutations but did not identify any variants which could explain the phenotype. Although it is possible that our approach may have missed a mutation, given that only 56% of the minimal deleted region was captured at ≥10X coverage in three patients, from our data the most likely explanation it that haploinsufficiency of one or more genes within the minimal deleted region is responsible for the hypoglycaemia. If this is true we would expect this aetiology to be associated with variable penetrance given that patients without hypoglycaemia and deletions over this region have been reported ^3^. This variable penetrance would be similar to what is observed with the gonadal dysgenesis phenotype where 46,XY patients with 9p24 deletions and normal male external genitalia have been reported ^3, 7^.

Interestingly, the deletions in four patients within our cohort do not overlap with the 3.5 Mb minimal deleted region defined by Faas *et al.* ^8^ and only two of our patients had a deletion which overlapped with the 300 kb critical region identified by Swinkels *et al.* ^3^. The majority of deletions in our patients were called from sequence data by savvyCNV ^8^ which maps breakpoints with an estimated accuracy of ±200 kb. Even including this margin of error, not all of our patients have deletions which overlap with either of the previously identified critical regions. This is in keeping with the 9p deletion syndrome being a genetically and phenotypically heterogeneous collection of overlapping syndromes.

In conclusion, our study identifies 9p deletions as an important cause of hypoglycaemia and refines the critical region for this phenotype to 7.2 Mb. Whilst we highlight potential candidate genes the genetic mechanism for the hypoglycaemia in our patients remains unknown. Further studies are required to investigate the cause of hyperinsulinism in these patients and in those with other copy number variant (CNV) syndromes which feature congenital hyperinsulinism such as Turner’s syndrome where the causative gene(s) have also not been definitively identified ^23, 24^. These large deletions can be screened for by targeted panels using an off-target CNV caller such as SavvyCNV ^12^.

## Data availability

### Underlying data

The genotype data could be used to identify individuals and so cannot be made openly available. Access to data is open only through collaboration. Requests for collaboration will be considered following an application to the Genetic Beta Cell Research Bank ( https://www.diabetesgenes.org/current-research/genetic-beta-cell-research-bank/). Contact by email should be directed to the Lead Nurse, Dr Bridget Knight ( b.a.knight@exeter.ac.uk).
