# Supplementary material for: Refinement of the critical genomic region for congenital hyperinsulinism in the Chromosome 9p deletion syndrome
Source: Wellcome Open Res. 2020 Aug 4;4:149. Originally published 2019 Oct 8. [Version 2] doi: 10.12688/wellcomeopenres.15465.2 (PMC7422856; doi:10.12688/wellcomeopenres.15465.2)
Supplement: Peer review history

# Peer review: Reviewer response for version 2

**DOI:** 10.21956/wellcomeopenres.17783.r39814

**Published:** 2020-08-18

**Authors:** Nicola Brunetti-Pierri

**Affiliations:** 1 Telethon Institute of Genetics and Medicine, Naples, Italy

Banjerjee and colleagues have adequately addressed all the previously raised issues.

Is the work clearly and accurately presented and does it cite the current literature?

Yes

If applicable, is the statistical analysis and its interpretation appropriate?

Not applicable

Are all the source data underlying the results available to ensure full reproducibility?

No source data required

Is the study design appropriate and is the work technically sound?

Yes

Are the conclusions drawn adequately supported by the results?

Partly

Are sufficient details of methods and analysis provided to allow replication by others?

Yes

Reviewer Expertise:

Medical Genetics

I confirm that I have read this submission and believe that I have an appropriate level of expertise to confirm that it is of an acceptable scientific standard.

# Peer review: Reviewer response for version 2

**DOI:** 10.21956/wellcomeopenres.17783.r39816

**Published:** 2020-08-11

**Authors:** Yao Yang

**Affiliations:** 1 Department of Genetics and Genomic Sciences, Icahn Institute for Genomics and Multiscale Biology, Icahn School of Medicine at Mount Sinai, New York, NY, USA

I have reviewed the revised manuscript and response. Authors have addressed my comments. I don’t have any further comments, and would suggest to accept this manuscript for indexing.

Is the work clearly and accurately presented and does it cite the current literature?

Yes

If applicable, is the statistical analysis and its interpretation appropriate?

Yes

Are all the source data underlying the results available to ensure full reproducibility?

No source data required

Is the study design appropriate and is the work technically sound?

Yes

Are the conclusions drawn adequately supported by the results?

Yes

Are sufficient details of methods and analysis provided to allow replication by others?

Yes

Reviewer Expertise:

NA

I confirm that I have read this submission and believe that I have an appropriate level of expertise to confirm that it is of an acceptable scientific standard.

# Peer review: Reviewer response for version 2

**DOI:** 10.21956/wellcomeopenres.17783.r39815

**Published:** 2020-08-11

**Authors:** Raphael Del Roio Liberatore Junior

**Affiliations:** 1 Pediatric Endocrinology‐Pediatric Diabetology and Metabology Section, Department of Pediatrics, School of Medicine of Ribeirão Preto, University of São Paulo, Ribeirao Preto, Brazil

It is ready to be indexed.

Is the work clearly and accurately presented and does it cite the current literature?

Yes

If applicable, is the statistical analysis and its interpretation appropriate?

Not applicable

Are all the source data underlying the results available to ensure full reproducibility?

Yes

Is the study design appropriate and is the work technically sound?

Yes

Are the conclusions drawn adequately supported by the results?

Yes

Are sufficient details of methods and analysis provided to allow replication by others?

Yes

Reviewer Expertise:

Pediatric Endocrinology

I confirm that I have read this submission and believe that I have an appropriate level of expertise to confirm that it is of an acceptable scientific standard.

# Peer review: Reviewer response for version 1

**DOI:** 10.21956/wellcomeopenres.16912.r38978

**Published:** 2020-06-24

**Authors:** Yao Yang

**Affiliations:** 1 Department of Genetics and Genomic Sciences, Icahn Institute for Genomics and Multiscale Biology, Icahn School of Medicine at Mount Sinai, New York, NY, USA

In the manuscript "Refinement of the critical genomic region for hypoglycemia in the Chromosome 9p deletion syndrome", authors studied 12 patients with chromosome 9p deletion syndrome who had a history of neonatal hypoglycemia. Study of such cases might be helpful to understand the disease mechanism. 

 The study was well designed and the manuscript was well structured and written. I will recommend to index the MS after comments below are addressed.

1. Figure 1 illustration of deletion seems not consistent with the deletion data shown in table 1. Either one should have error. 
2. Did author test those genes associated with hypoglycemia to rule out pathogenic variants on those genes for these cases?
3. The NGS data for the sequencing didn't achieve enough depth and coverage to rule out the third possible mechanism mentioned by author: unmasking a recessive mutation. I understand authors already discussed a little bit about his, but a more comprehensive analysis of NGS data with enough depth and coverage will strength the conclusion of this paper. However, I wouldn't say this is necessary to index. 

Is the work clearly and accurately presented and does it cite the current literature?

Yes

If applicable, is the statistical analysis and its interpretation appropriate?

Yes

Are all the source data underlying the results available to ensure full reproducibility?

No source data required

Is the study design appropriate and is the work technically sound?

Yes

Are the conclusions drawn adequately supported by the results?

Yes

Are sufficient details of methods and analysis provided to allow replication by others?

Yes

Reviewer Expertise:

Clinical Molecular Genetics, Bioinformatics, NGS

I confirm that I have read this submission and believe that I have an appropriate level of expertise to confirm that it is of an acceptable scientific standard, however I have significant reservations, as outlined above.

# Response

**Published:** 2020-07-24

**Authors:** Sarah Flanagan

**Affiliations:** University of Exeter, UK

**In the manuscript "Refinement of the critical genomic region for hypoglycemia in the Chromosome 9p deletion syndrome", authors studied 12 patients with chromosome 9p deletion syndrome who had a history of neonatal hypoglycemia. Study of such cases might be helpful to understand the disease mechanism.**

****

**The study was well designed and the manuscript was well structured and written. I will recommend to index the MS after comments below are addressed.**

****

**1.    Figure 1 illustration of deletion seems not consistent with the deletion data shown in table 1. Either one should have error.**

 We thank the reviewer for their appraisal of our manuscript. We are grateful for highlighting the discrepancies between table 1 and figure 1. A new version of figure 1 has now been uploaded which includes patient 3 (omitted by mistake in the previous version). The tables and figure have now bee double checked.

**2.    Did author test those genes associated with hypoglycemia to rule out pathogenic variants on those genes for these cases?**

 We apologise that this was unclear. We had excluded all known causes of congenital hyperinsulinism in 9 of the 12 patients. In the three remaining patients samples were unavailable to targeted next-generation sequencing. We have updated the methods to reflect this point.

**3.    The NGS data for the sequencing didn't achieve enough depth and coverage to rule out the third possible mechanism mentioned by author: unmasking a recessive mutation. I understand authors already discussed a little bit about his, but a more comprehensive analysis of NGS data with enough depth and coverage will strength the conclusion of this paper. However, I wouldn't say this is necessary to index**.

 We agree with the reviewer that it has not been possible to rule out a missed recessive mutation given the depth/coverage of the sequencing, which we hope is reflected in the text of our discussion.

# Peer review: Reviewer response for version 1

**DOI:** 10.21956/wellcomeopenres.16912.r38980

**Published:** 2020-06-18

**Authors:** Nicola Brunetti-Pierri

**Affiliations:** 1 Telethon Institute of Genetics and Medicine, Naples, Italy

Banjerjee and colleagues report 12 patients with 9p deletions and hypoglycemia. They also defined the minimally deleted interval trying to pinpoint the gene responsible for the metabolic defect.

 The paper is well written and provides novel information. However, there are several issues that require attention: 

1. There are discrepancies between Fig. 1 and Table 1:a) Patient 3 is reported to have a 17.6 Mb deletion on table 1 (larger than the deletion of patient 2) but according to figure 1, the deletion is smaller than patient 2.b) Deletion size is similar between patients 6-7-8-9 and yet patient 9 is shown in fig-1 to have a larger deletion. Patient 4 and 5 deletions should be similar in size according to the table.
2. In Fig. 1: patient 12 is not shown. Why there is a single base for 9/10?
3. Table 1: why there is a ‘not applicable’ for the age of hypoglycemia remission of patient 3? Is it not available?
4. Indicate on the table that the glucose levels were at the time of onset of the hypoglycemia.
5. Please add whether each patient was AGA, SGA or LGA according to their birth weight and gestational age.
6. Dose of diazoxide is not included in all cases.
7. Please change age at diagnosis to ‘onset’ to make clear it refers to the hypoglycemia.
8. Fig. 1 should include the size of the deletions and an Ensembl/USC snapshot of the genes included in the minimally deleted interval.
9. Is any of the genes in the interval imprinted? Imprinting would be a fourth mechanism that the authors can include in the discussion. Moreover, there are defects of imprinted genes known to cause hyperinsulinism (e.g., 11p15 region) and neonatal diabetes.
10. Discuss whether haploinsufficiency for the genes in the interval are responsible for any known syndromes and whether these syndromes have been associated to hypoglycemia.
11. Can the authors estimate the frequency of the hypoglycemia among all reported cases of 9p deletions? This estimate would help understanding whether mutations on the undeleted allele might be implicated. Given the apparently higher frequency of hypoglycemia in 9p deletion cases, one would predict that the underlying mechanism for hypoglycemia it cannot be due to unmasking of a recessive allele.
12. Add DECIPHER ID for the case included on fig. 1.

Is the work clearly and accurately presented and does it cite the current literature?

Yes

If applicable, is the statistical analysis and its interpretation appropriate?

Not applicable

Are all the source data underlying the results available to ensure full reproducibility?

No source data required

Is the study design appropriate and is the work technically sound?

Yes

Are the conclusions drawn adequately supported by the results?

Partly

Are sufficient details of methods and analysis provided to allow replication by others?

Yes

Reviewer Expertise:

Medical Genetics

I confirm that I have read this submission and believe that I have an appropriate level of expertise to confirm that it is of an acceptable scientific standard, however I have significant reservations, as outlined above.

# Response

**Published:** 2020-07-24

**Authors:** Sarah Flanagan

**Affiliations:** University of Exeter, UK

**Banerjee and colleagues report 12 patients with 9p deletions and hypoglycemia. They also defined the minimally deleted interval trying to pinpoint the gene responsible for the metabolic defect. The paper is well written and provides novel information. However, there are several issues that require attention**

**1.    There are discrepancies between Fig. 1 and Table 1:a) Patient 3 is reported to have a 17.6 Mb deletion on table 1 (larger than the deletion of patient 2) but according to figure 1, the deletion is smaller than patient 2.b) Deletion size is similar between patients 6-7-8-9 and yet patient 9 is shown in fig-1 to have a larger deletion. Patient 4 and 5 deletions should be similar in size according to the table. Fig. 1: patient 12 is not shown. Why there is a single base for 9/10?**

 We thank the reviewer for their thorough review of our manuscript and for highlighting the discrepancies between the table and the figure. We had mistakenly uploaded a previous version of figure 1 for peer review which had not included patient 3, this patient had been included in the table. A new figure has been uploaded and the data double checked.

   

**2.    Table 1: why there is a ‘not applicable’ for the age of hypoglycemia remission of patient 3? Is it not available?**

 The table has been updated to show that the child had normal glycaemia at the age of 7 months.

   

**3.    Indicate on the table that the glucose levels were at the time of onset of the hypoglycemia.**

 The table has been updated as requested.

   

**5.    Please add whether each patient was AGA, SGA or LGA according to their birth weight and gestational age.**

 The birth weight standard deviation score for each patient has been added to table 1. We would prefer to add SDS scores than AGA/SGA/LDA. 

   

**6.    Dose of diazoxide is not included in all cases.**

 We had included all of the information that we have available to us regarding diazoxide dose. When the dose is unavailable we had noted this in the manuscript. 

   

**7.    Please change age at diagnosis to ‘onset’ to make clear it refers to the hypoglycemia.**

 The table has been updated as requested.

   

**8.    Fig. 1 should include the size of the deletions and an Ensembl/USC snapshot of the genes included in the minimally deleted interval.**

 We have added the size of the minimal deleted region to the figure as requested. The size of each of the individual deletions is recorded in table 1. We felt that annotating them to the figure reduced clarity. Due to the  minimal deleted region containing 38 protein coding genes (listed in table 2) we also felt that these would be difficult to include in figure 1 in a way that could be clearly read. We have not updated the legend to help highlight that this information is available within the tables.

   

**9.    Is any of the genes in the interval imprinted? Imprinting would be a fourth mechanism that the authors can include in the discussion. Moreover, there are defects of imprinted genes known to cause hyperinsulinism (e.g., 11p15 region) and neonatal diabetes.**

 We agree that disruption of imprinted genes is a possible mechanism of disease in individuals with large CNVs. We had checked the genes in the minimal deleted region but none are known to be imprinted (Baran et al The landscape of genomic imprinting across diverse adult human tissues. Genome Res. 2015 Jul;25(7):927-36). We have now added a sentence to the discussion to reflect this point.

**10.    Discuss whether haploinsufficiency for the genes in the interval are responsible for any known syndromes and whether these syndromes have been associated to hypoglycemia.**

 Further information to highlight which of the genes within the deleted region are reported to cause disease when disrupted have now been included in table 2. With the exception of *SLC1A1* none of these genes have been associated with hypoglycaemia. The OMIM record for *SLC1A1* list fasting hypoglycaemia as a feature however this information is taken from a case report published in 1974 (Teijema et al: Dicarboxylic aminoaciduria: An inborn error of glutamate and aspartate transport with metabolic implications, in combination with a hyperprolinemia, Metabolism 23:115, 1974) prior to discovery of the *SLC1A1* gene (Bailey et al Loss-of-function mutations in the glutamate transporter *SLC1A1* cause human dicarboxylic aminoaciduria. J. Clin. Invest. 121: 446-453, 2011). 

**11.    Can the authors estimate the frequency of the hypoglycemia among all reported cases of 9p deletions? This estimate would help understanding whether mutations on the undeleted allele might be implicated. Given the apparently higher frequency of hypoglycemia in 9p deletion cases, one would predict that the underlying mechanism for hypoglycemia it cannot be due to unmasking of a recessive allele.**

 We do not believe that it would be accurate to offer a prediction on the frequency of hypoglycaemia in the 9p deletion syndrome based on published cases as this does not provide an unbiased cross-sectional cohort. We agree with the reviewer that unmasking of a recessive allele is unlikely, we hope we have addressed this point adequately in the discussion. 

   

**12.    Add DECIPHER ID for the case included on fig. 1.**

 The ID for the Decipher case has now been included on figure 1.

# Peer review: Reviewer response for version 1

**DOI:** 10.21956/wellcomeopenres.16912.r37375

**Published:** 2019-12-19

**Authors:** Raphael Del Roio Liberatore Junior

**Affiliations:** 1 Pediatric Endocrinology‐Pediatric Diabetology and Metabology Section, Department of Pediatrics, School of Medicine of Ribeirão Preto, University of São Paulo, Ribeirao Preto, Brazil

The authors studied 12 patients with 9p deletion syndrome who had hypoglycaemia in the neonatal period in an attempt to find a genetic cause to explain hypoglycaemia.

 Hypoglycaemia is a very uncommon symptom of 9p deletion syndrome and hyperinsulinism has recently been described in patients with 9p deletion syndrome as a cause for hypoglycaemia in these patients. Thus, the study proposal is appropriated and justified.

 Regarding clinical aspects, the authors report that patients 03 and 12, although presenting hypoglycaemia, did not have confirmation of hyperinsulinism. In addition, these 2 patients did not require treatment.

 The experimental approach is quite elegant and complete, with the aim of finding a genetic cause for hypoglycaemia in 9p deletion syndrome patients. Unfortunately, this cause was not found.

 However, here are some considerations:

1. In the fourth paragraph of the introduction, the authors report that in 55% of cases of congenital hyperinsulinism, no genetic causes are found. Thus, I believe that major contribution of this research could be that candidate genes present in the lost region of chromosome 9 do not explain the occurrence of congenital hyperinsulinism. I believe that patients 03 and 12 may be excluded from the sample and the title could may be changed to: Refinement of the critical genomic region for congenital hyperinsulinism in chromosome 9p deletion syndrome.
2. Still regarding patients 03 and 12, it is not clear what was the cause of the episode(s) of hypoglycaemia. These patients may have hypopituitarism, especially patient 03, who may have changes in the medial line.
3. It was not clear in the manuscript if the genes known to be related to congenital hyperinsulinism have being studied.
4. Interestingly, although 9p deletion syndrome is described as gonadal dysgenesis and sex reverse, only patients 1, 4, and 8 had genital alterations. I don't know if the authors paid attention to this fact.

 I would be absolutely satisfied with the answer to these considerations that even reduce the  importance and elegance of the present study.

Is the work clearly and accurately presented and does it cite the current literature?

Yes

If applicable, is the statistical analysis and its interpretation appropriate?

Not applicable

Are all the source data underlying the results available to ensure full reproducibility?

Yes

Is the study design appropriate and is the work technically sound?

Yes

Are the conclusions drawn adequately supported by the results?

Yes

Are sufficient details of methods and analysis provided to allow replication by others?

Yes

Reviewer Expertise:

Pediatric Endocrinology

I confirm that I have read this submission and believe that I have an appropriate level of expertise to confirm that it is of an acceptable scientific standard, however I have significant reservations, as outlined above.

# Response

**Published:** 2020-07-24

**Authors:** Sarah Flanagan

**Affiliations:** University of Exeter, UK

**The authors studied 12 patients with 9p deletion syndrome who had hypoglycaemia in the neonatal period in an attempt to find a genetic cause to explain hypoglycaemia.**

****

**Hypoglycaemia is a very uncommon symptom of 9p deletion syndrome and hyperinsulinism has recently been described in patients with 9p deletion syndrome as a cause for hypoglycaemia in these patients. Thus, the study proposal is appropriated and justified.**

****

**Regarding clinical aspects, the authors report that patients 03 and 12, although presenting hypoglycaemia, did not have confirmation of hyperinsulinism. In addition, these 2 patients did not require treatment.**

****

**The experimental approach is quite elegant and complete, with the aim of finding a genetic cause for hypoglycaemia in 9p deletion syndrome patients. Unfortunately, this cause was not found.**

****

**1.    In the fourth paragraph of the introduction, the authors report that in 55% of cases of congenital hyperinsulinism, no genetic causes are found. Thus, I believe that major contribution of this research could be that candidate genes present in the lost region of chromosome 9 do not explain the occurrence of congenital hyperinsulinism. I believe that patients 03 and 12 may be excluded from the sample and the title could may be changed to: Refinement of the critical genomic region for congenital hyperinsulinism in chromosome 9p deletion syndrome.**

 We thank the reviewer for their constructive appraisal of our manuscript. We would like to keep patients 03 and 12 within the cohort as we believe that it is important to report that there may be variability in the biochemical hypoglycaemic screens in these patients. That said, we agree that there is overwhelming evidence that these deletions result in hyperinsulinemic hypoglycaemia in the majority of patients and as such we have now altered the title as suggested to reflect this point. 

**2.    Still regarding patients 03 and 12, it is not clear what was the cause of the episode(s) of hypoglycaemia. These patients may have hypopituitarism, especially patient 03, who may have changes in the medial line.**

 Thank you for highlighting this point; cortisol deficiency was excluded in all patients clinically and biochemically at diagnosis and no patients had evidence of growth failure.  The paper has now been updated to reflect this.

**3.    It was not clear in the manuscript if the genes known to be related to congenital hyperinsulinism have being studied.**

 We apologise that this was unclear. We have now updated the methodology section to clarify which patients within our cohort underwent targeted next-generation sequencing.

**4.    Interestingly, although 9p deletion syndrome is described as gonadal dysgenesis and sex reverse, only patients 1, 4, and 8 had genital alterations. I don't know if the authors paid attention to this fact**.  

 ​​​​​​​We agree it is interesting that of the 7 individuals in our cohort with a 46XY karyotype only 3 had gonadal dysgenesis. The variable penetrance with this phenotype is included within the discussion.
